# Identification of genomic regions controlling spikelet degeneration under *FRIZZLE PANICLE* (*FZP*) defect genetic background in rice

**DOI:** 10.1038/s41598-024-63362-8

**Published:** 2024-05-30

**Authors:** Sheng-Shan Wang, Pei-Hua Tsai, Shu-Fang Cheng, Rong-Kuen Chen, Kai-Yi Chen

**Affiliations:** 1 Tainan District Agricultural Research and Extension Station, No. 70, Muchang, Xinhua, Tainan, 71246 Taiwan; 2 Department of Agronomy, National Taiwan University, No. 1, Sec. 4, Roosevelt Rd., Taipei, 10617 Taiwan

**Keywords:** *Oryza sativa*, Axillary branch, Panicle architecture, Developmental biology, Genetics, Plant sciences

## Abstract

The *FZP* gene plays a critical role in the formation of lateral branches and spikelets in rice panicle architecture. This study investigates the *qSBN7* allele, a hypomorphic variant of *FZP*, and its influence on panicle architectures in different genetic backgrounds. We evaluated two backcross inbred lines (BILs), BC5_TCS10^*sbn*^ and BC3_TCS10^*sbn*^, each possessing the homozygous *qSBN7* allele but demonstrating differing degrees of spikelet degeneration. Our analysis revealed that BC5_TCS10^*sbn*^ had markedly low *FZP* expression, which corresponded with an increase in axillary branches and severe spikelet degeneration. Conversely, BC3_TCS10^*sbn*^ exhibited significantly elevated *FZP* expression, leading to fewer secondary and tertiary branches, and consequently decreased spikelet degeneration. Compared to BC5_TCS10^*sbn*^, BC3_TCS10^*sbn*^ carries three additional chromosomal substitution segments from its donor parent, IR65598-112-2. All three segments significantly enhance the expression of *FZP* and reduce the occurrence of tertiary branch and spikelet degeneration. These findings enhance our understanding of the mechanisms regulating *FZP* and aid rice breeding efforts.

## Introduction

The number of spikelets per panicle is a crucial factor in determining rice grain yield. This number varies depending on various components of a rice panicle, such as panicle length, the number of primary, secondary, and tertiary branches, and the number of lateral and terminal spikelets. These variations result from subtle changes in panicle architecture during development. During the reproductive stage, the apical meristem of the rice shoot evolves into the rachis meristem, which is responsible for producing axillary meristems that develop into primary branches. Subsequently, the basal meristem of these primary branches becomes secondary branches, while the upper lateral and terminal meristems directly form spikelet meristems. All meristems, including the lateral and terminal meristems of the secondary branches, develop into spikelets. Some cultivars with high spikelet numbers may develop tertiary branches from the basal meristem of secondary branches. Eventually, meristems of these tertiary branches also transform into spikelets^1,2^. Researchers have identified several genes associated with panicle architecture, such as *APO1*, *APO2*, *FZP*, *Gn1a*, *IPA1*/*WFP*, *LAX1*, *LAX2*, *RCN1*, *RCN2* and *TAW1*^3–12^. In addition to panicle architecture, spikelet degeneration is another factor affecting the number of spikelets per panicle. Spikelet degeneration results from genetic defects in specific genes, including *ASA*, *DPS1*, *FZP*, *OsALMT7*, *PAA3*, *TSD1*, *TUTOU1* and *TUTOU2*^5,13–19^, as well as from resource-limited conditions such as shading, drought, and heat stress^20–23^.

Our previous study identified a beneficial, hypomorphic allele of *FZP* under the *qSBN7* quantitative trait loci (QTL), which increases the number of spikelets per panicle and grain yield^24^. *FZP* encodes an AP2/ERF transcription factor, regulates the balance between branches and spikelet meristem formation^5,25–28^. A nonfunctional *FZP* variant leads to an exceptionally high number of secondary and higher-order branches, resulting in severe spikelet degeneration^5^. By contrast, several *FZP* hypomorphic alleles increase the number of secondary branches and spikelets without apparent degeneration, thus demonstrating their value for the breeding of elite rice varieties^24,29,30^. Nevertheless, introgression of the same *FZP* hypomorphic allele (*qSBN7*) into the *indica* rice variety TCS10 resulted in severe panicle degeneration and aborted spikelets^24^. The current study aims to gain further insights into the impact of *FZP* on panicle architecture under different genetic backgrounds.

## Results

### Phenotypic characterization of the BC5_TCS10^*sbn*^

**Figure 1:**
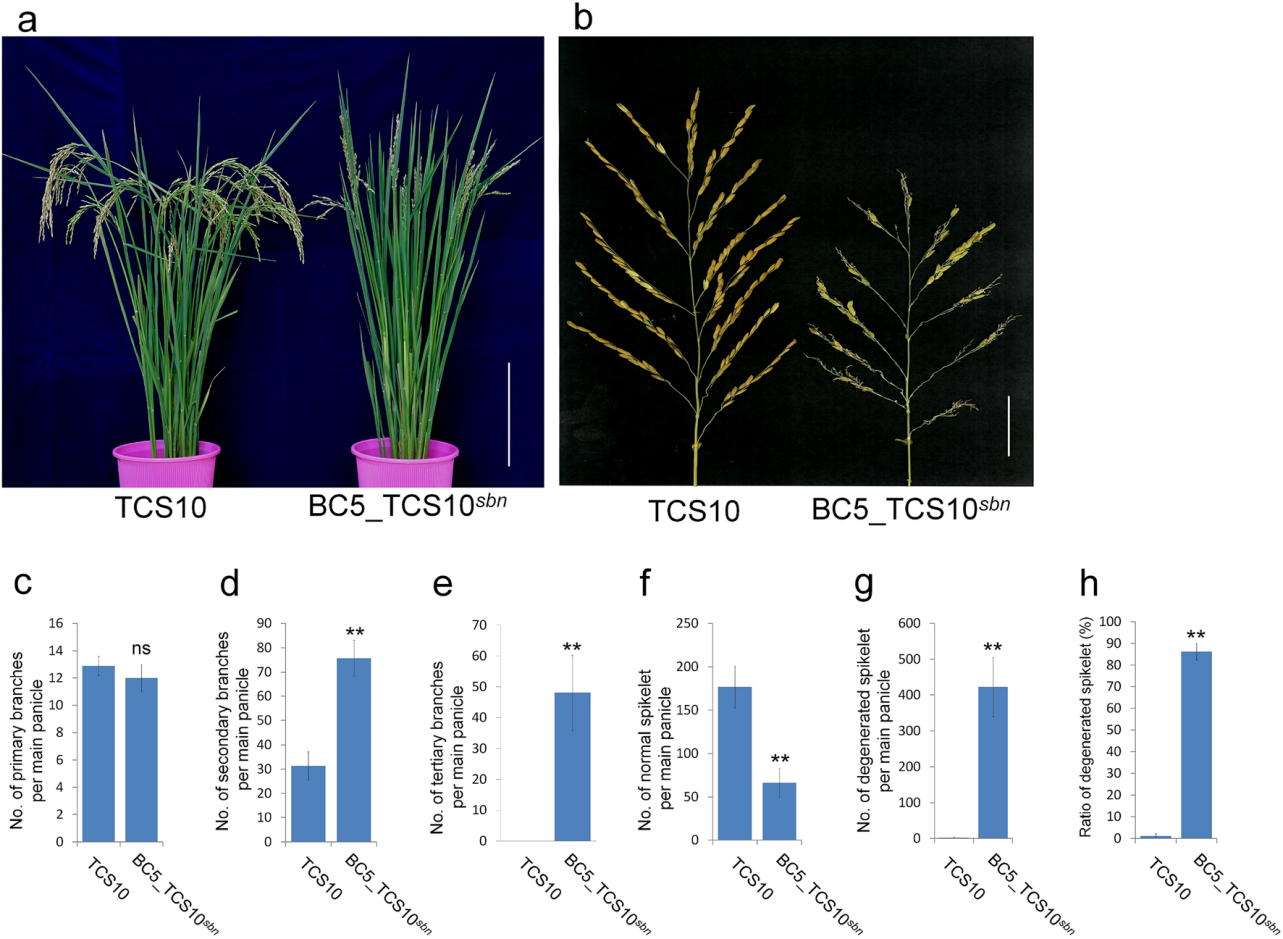
Phenotypic characterization of TCS10 and BC5_TCS10^*sbn*^. (**a**) Overall plant morphology of TCS10 and BC5_TCS10^*sbn*^. Scale bar = 20 cm. (**b**) Structure of panicles from TCS10 and BC5_TCS10^*sbn*^. Scale bar = 4 cm. (**c**–**h**) Comparison of number of primary, secondary, and tertiary branches and number of normal and degenerated spikelets per main panicle, including the ratio of degenerated spikelets for TCS10 and BC5_TCS10^*sbn*^. Values in (**c**–**h**) are means ± SD. Student’s *t* test was used to examine *p* values. ** indicates significance at 1% level; ns denotes not significant.

BC5_TCS10^*sbn*^, a BC5 backcross inbred line (BIL) which was developed using the IR65598-112-2 rice variety as the donor parent, and TCS10 as the recurrent parent. To elucidate the phenotypic characteristics of BC5_TCS10^*sbn*^, we compared its agronomic traits during the vegetative and reproductive stages with TCS10. The plant morphology of BC5_TCS10^*sbn*^ closely resembled that of TCS10 (Fig. 1a); however, the panicle of BC5_TCS10^*sbn*^ exhibited severe degeneration (Fig. 1b and Supplementary Fig. S1). We observed no significant difference in the primary branch number per panicle between BC5_TCS10^*sbn*^ and TCS10 (Fig. 1c). Nevertheless, BC5_TCS10^*sbn*^ displayed higher number of secondary and tertiary branches per panicle (Fig. 1d,e). Compared with TCS10, the panicles of BC5_TCS10^*sbn*^ bore only 66 normal spikelets and exhibited severe degeneration in spikelets (Fig. 1f–h).

**Figure 2:**
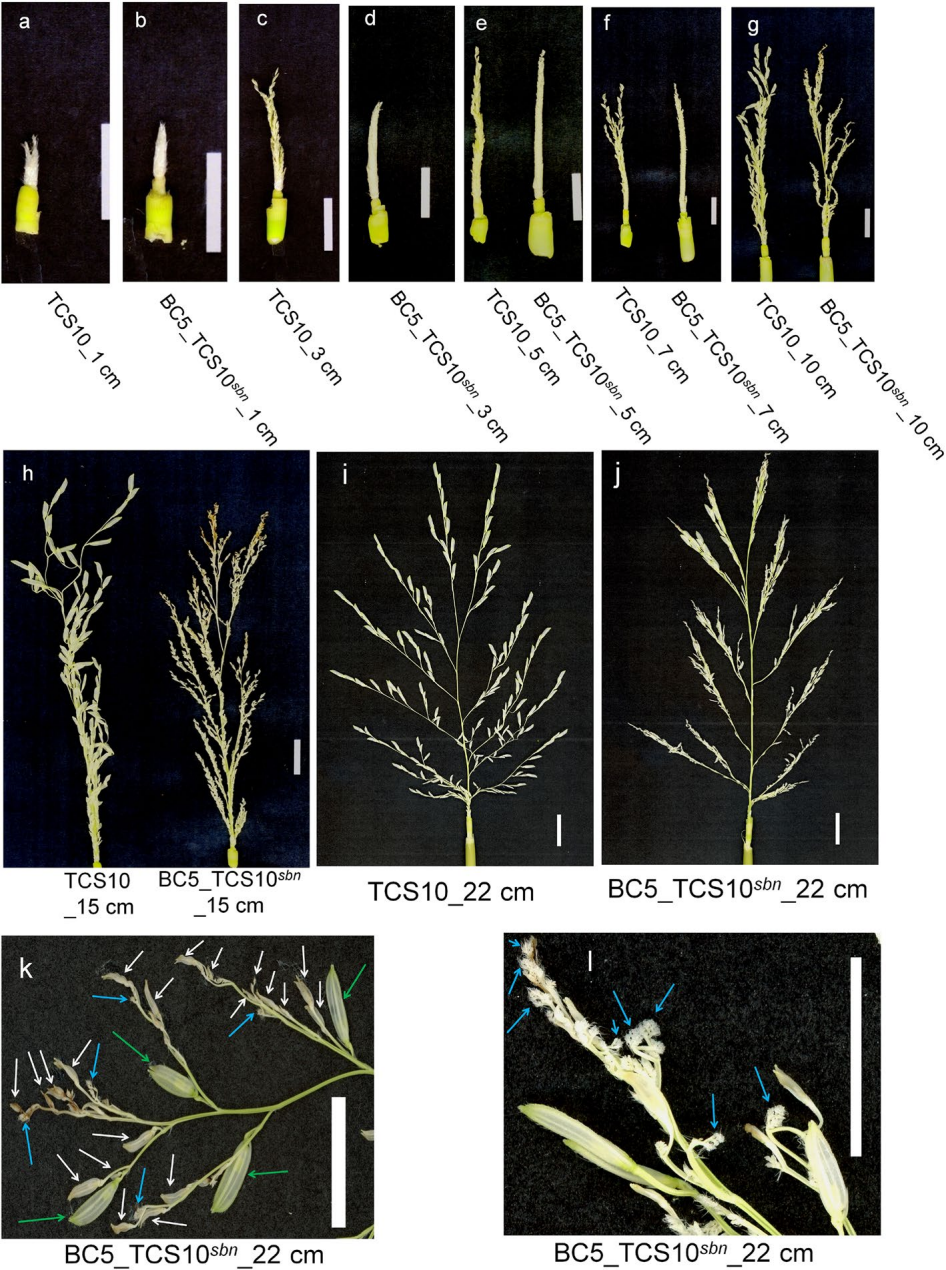
Panicle morphology of TCS10 and BC5_TCS10^*sbn*^ at different developmental stages. (**a**–**j**) Panicle morphology of TCS10 and BC5_TCS10^*sbn*^ from 1–22 cm. Scale bar = 1.5 cm. (**k**) Normal spikelets (green arrows),degenerated spikelets (white arrows) and continuous bract-like structures (blue arrows) were observed in the 22 cm panicle of BC5_TCS10^*sbn*^. Scale bar = 1.0 cm. (**l**) Continuous bract-like structures (blue arrows) were observed in the 22 cm panicle of BC5_TCS10^*sbn*^. Scale bar = 1.0 cm.

**Figure 3:**
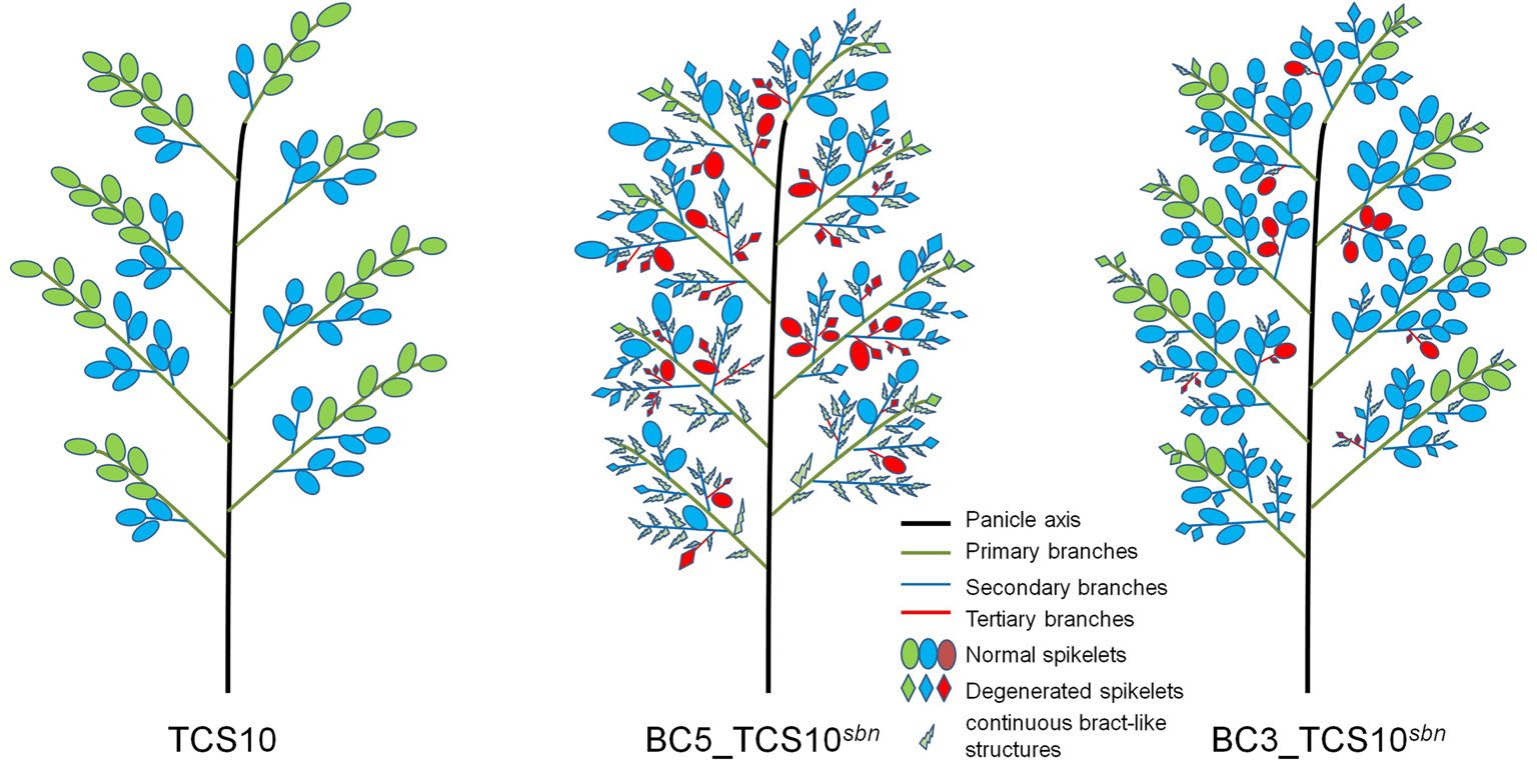
Schematic of rice panicle structures in TCS10, BC5_TCS10^*sbn*^, and BC3_TCS10^*sbn*^.

To determine the timing and location of degeneration, we investigated the development of panicles from 1 to 22 cm. In TCS10 panicles, spikelets were observed to form and rapidly elongate from 3 to 22 cm (Fig. 2a,c,e–i). Conversely, in BC5_TCS10^*sbn*^ panicles, more than half of the spikelets were replaced by numerous continuous bract-like structures (Fig. 2l). The remaining spikelets in BC5_TCS10^*sbn*^ demonstrated a gradual delay in development from 3 to 15 cm (Fig. 2b,d–h). In BC5_TCS10^*sbn*^ panicles, only a few spikelets positioned at the basal or the apical of secondary and tertiary branches rapidly elongated from 15 to 22 cm and developed into survival spikelets after heading (Fig. 2j,k and Supplementary Fig. S1). These findings indicated that the panicle architecture of BC5_TCS10^*sbn*^ closely resembled that of severe *fzp* mutants (*fzp-3*, *fzp-14* and *abp1*) identified in previously studies. The rice panicle structure observed in TCS10 and BC5_TCS10^*sbn*^ is illustrated in Fig. 3.

### Development of BC3_TCS10^*sbn*^ line with slightly degenerated panicle

**Figure 4:**
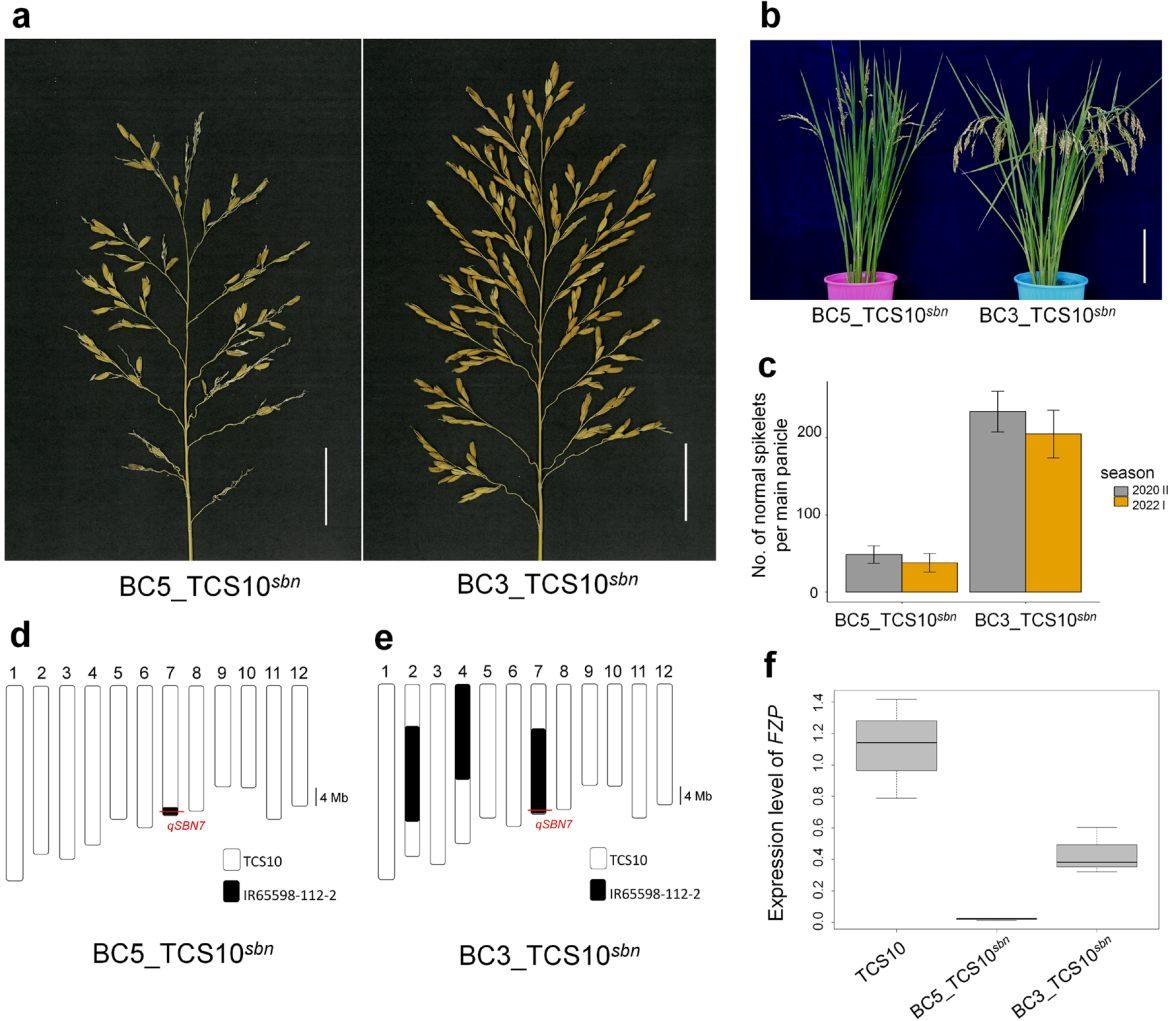
Phenotypic characterization and *FZP* expression in BC3_TCS10^*sbn*^ and BC5_TCS10^*sbn*^. (**a**) Panicle morphology of two backcross inbred lines (BILs). Scale bar = 4 cm. (**b**) Plant morphology of two BILs. Scale bar = 20 cm. (**c**) Number of normal spikelets per main panicle in the two BILs across two crop seasons. (**d**, **e**) Graphical genotypes of the two BILs. White squares: homozygous chromosomal segments of TCS10; black squares: homozygous chromosomal segment of IR65598-112–2. (**f**) Transcript levels of *FZP* in TCS10, BC5_TCS10^*sbn*^ and BC3_TCS10^*sbn*^ at the 1-mm panicle stage.

*qSBN7* allele was introgressed from a new plant type variety, IR65598-112-2, which presents high spikelet number without significant degradation. In order to determine the genetic factors responsible for the differences in degeneration between BC5_TCS10^*sbn*^ and IR65598-112-2, a BC_3_-derived BIL (BC3_TCS10^*sbn*^) with slightly degraded panicles from the same parents as BC5_TCS10^*sbn*^ were developed (Fig. 4a,b). Unlike BC5_TCS10^*sbn*^, BC3_TCS10^*sbn*^’s panicles harbored almost three times the number of normal spikelets, ranging between 190 and 220 (Fig. 4a,c). Furthermore, we evaluated five yield components between TCS10 and BC3_TCS10^*sbn*^. BC3_TCS10^*sbn*^ exhibited a significant increase in secondary branches and normal spikelets per panicle, while displaying a decrease in 1000-grain weight compared to TCS10 (Supplementary Figure S2). However, no significant difference in the percentage of filled grains and panicle weight was observed between two lines (Supplementary Figure S2).

To determine the genetic differences between BC5_TCS10^*sbn*^ and BC3_TCS10^*sbn*^*, *we conducted a whole-genome survey of IR65598-112-2, TCS10, BC5_TCS10^*sbn*^*, *and BC3_TCS10^*sbn*^ using next-generation sequencing. Our results indicated that BC5_TCS10^*sbn*^ only carried an introgression segment (28.30–29.69 Mb) from IR65598-112-2 on chromosome 7, with approximately 99.7% of the recurrent parent genome recovered (Fig. 4d). Conversely, BC3_TCS10^*sbn*^ had three substituted segments from IR65598-112-2, specifically IG2 (10.33–29.32 Mb) on chromosome 2, IG4 (0–21.21 Mb) on chromosome 4, and IG7 (9.08–29.69 Mb) on chromosome 7, resulting in an 84.1% recovery of BC3_TCS10^*sbn*^ (Fig. 4e). This suggests that the genes contributing to the phenotypic differences between the two BILs likely reside within these segments.

### Evaluation of the causal factors leading to phenotypic differences between BC5_TCS10^*sbn*^ and BC3_TCS10^*sbn*^

There are two potential explanations for the variation between two BILs. One explanation is that BC3_TCS10^*sbn*^ contains genes that control *FZP* and suppress the growth of secondary and tertiary branches. The other explanation is that BC3_TCS10^*sbn*^ carries genes that enhance photosynthesis or carbohydrate translocation, leading to an increase in the number of survival spikelets. To understand which explanation is more realistic, we conducted quantitative PCR (qPCR) to assess the transcript levels of *FZP* in TCS10, BC5_TCS10^*sbn*^, and BC3_TCS10^*sbn*^ at the 1-mm panicle stage. Our findings reveal that BC5_TCS10^*sbn*^ exhibited a 98.2% decrease in *FZP* expression compared with TCS10 (Fig. 4f), whereas BC3_TCS10^*sbn*^ exhibited intermediate *FZP* expression levels, distinct from TCS10 and BC5_TCS10^*sbn*^ (Fig. 4f). A further comparison of panicle characteristics between BC5_TCS10^*sbn*^ and BC3_TCS10^*sbn*^ revealed that BC3_TCS10^*sbn*^ had more normal spikelets and primary branches than BC5_TCS10^*sbn*^, but fewer secondary branches, tertiary branches, and the ratio of degenerated spikelet (Table 1). This indicates that BC3_TCS10^*sbn*^ might possess genes that enhance *FZP* expression, thereby limiting axillary differentiation and the occurrence of degenerate spikelets.

**Table 1:** Comparison of panicle-related and source-related traits between BC5_TCS10^*sbn*^ and BC3_TCS10^*sbn*^.

Traits^1^	BC5_TCS10^*sbn*^	BC3_TCS10^*sbn*^	*P*-value
Primary branch number	12.0 ± 1.0	16.0 ± 0.9	< 0.01
Secondary branch number	75.1 ± 7.4	66.9 ± 10.1	< 0.01
Tertiary branch number	48.1 ± 12.1	4.4 ± 4.3	< 0.01
No. of normal spikelet	66.3 ± 16.8	250.1 ± 30.9	< 0.01
Ratio of degenerated spikelet (%)	86.2 ± 3.8	18.1 ± 8.7	< 0.01
Culm diameter (mm)	1.96 ± 0.17	2.33 ± 0.13	< 0.01
Chlorophyll content	48.63 ± 2.2	48.72 ± 2.6	0.913
Flag leaf length (cm)	36.0 ± 3.6	35.0 ± 3.8	0.45
Flag leaf width (mm)	17.0 ± 0.8	16.5 ± 0.8	0.09

The *p*-value indicates the significance of the difference between BC5_TCS10^*sbn*^ and BC3_TCS10^*sbn*^ based on Student’s *t*-test.

We also assessed whether source-related traits varied between BC5_TCS10^*sbn*^ and BC3_TCS10^*sbn*^. A comparative analysis of various traits, including culm diameter, flag leaf length, flag leaf width, and chlorophyll content of the flag leaf, revealed a significant difference only in culm diameter between the two BILs (Table 1). This finding suggests that BC3_TCS10^*sbn*^ may possess genes influencing culm diameter, which may influence carbohydrate allocation and contribute to the production of more regular spikelets. Thus, increased in *FZP* expression and changed in culm diameter are likely to jointly contribute to the reduced number of degenerate spikelets in BC3_TCS10^*sbn*^.

### Detection of genomic regions associated with degenerated spikelets traits between two BILs

**Figure 5:**
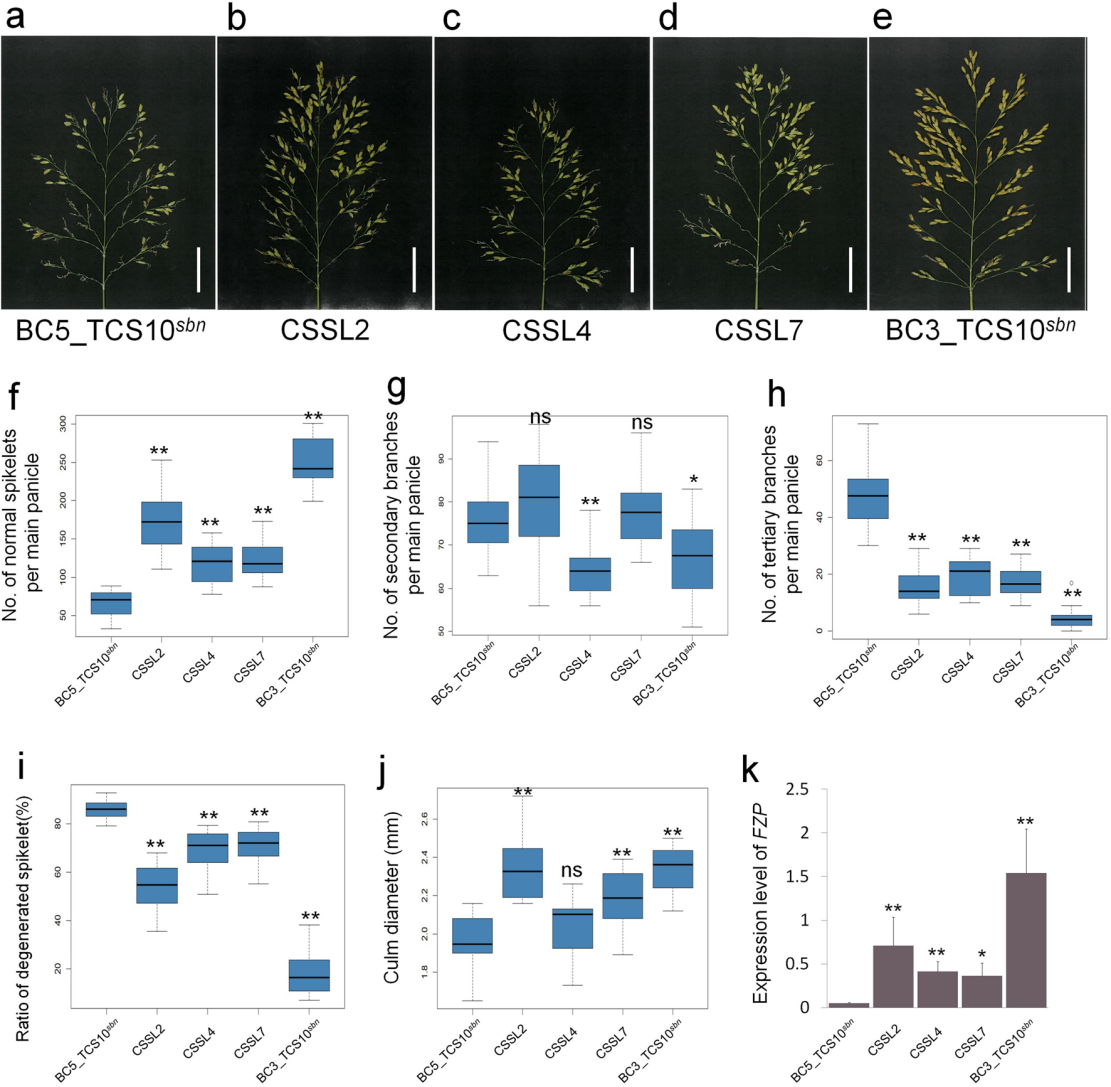
Panicle characterization and *FZP* expression levels in BC5_TCS10^*sbn*^, BC3_TCS10^*sbn*^, and three CSSLs (chromosome segment substitution lines). (**a–e**) Panicle morphology of BC5_TCS10^*sbn*^, CSSL2, CSSL4, CSSL7 and BC3_TCS10^*sbn*^. Scale bar = 4 cm. (**f–j**) Comparative analyses of BC5_TCS10^*sbn*^, BC3_TCS10^*sbn*^, and the three CSSLs in terms of normal spikelet number, secondary branch number, tertiary branch number per main panicle, ratio of degenerated spikelets, and culm diameter. (**k**) *FZP* transcript levels in BC5_TCS10^*sbn*^, BC3_TCS10^*sb*^, and three CSSLs. ns indicates no significant difference; *, ** indicates significant differences compared with BC5_TCS10^*sbn*^ at α = 0.05, 0.01, respectively, based on the Dunnett’s multiple range test.

To identify genomic regions related to degenerated spikelet traits, we developed three chromosome segment substitution lines (CSSLs). Each line contained homozygous substituted segments from IR65598-112-2. We analyzed these lines for genomic regions related to degenerated spikelet traits and performed qPCR to examine *FZP* transcript levels in BC5_TCS10^*sbn*^, BC3_TCS10^*sbn*^, and the three CSSLs. Our results demonstrated that each CSSL exhibited an increased number of normal spikelets, fewer tertiary branches, and a lower ratio of degenerated spikelets compared with BC5_TCS10^*sbn*^ (Fig. 5a–i). Additionally, *FZP* expression was higher in all three CSSLs than in BC5_TCS10^*sbn*^ (Fig. 5k). This expression exhibited a strong positive correlation with the number of normal spikelets per panicle and a negative correlation with the ratio of degenerated spikelets. These results suggest that the substituted segments from IR65598-112-2 in each CSSL could interact with *FZP* to repress the formation of tertiary branches. Furthermore, CSSL2 and CSSL7 exhibited significantly larger culm diameters in BC3_TCS10^*sbn*^ (Fig. 5j). This observation indicates that CSSL2 and CSSL7 contain QTLs capable of increasing culm diameter, which in turn may influence the degree of spikelet degeneration.

## Discussion

*FZP* is an essential gene that regulates rice panicle architecture. To date, researchers have identified 21 *FZP* alleles in rice^5,24–35^. In general, null alleles in *FZP* mutants lead to the formation of higher-order rachis branches rather than spikelets, leading to an absence of normal spikelets^5,25,31,32,35^. Severe hypomorphic mutants of *FZP* exhibit an increased number of secondary and tertiary panicle branches and produce partially normal spikelets due to their partial functionality. However, these mutants often have various degenerated or defective spikelets^5,26–28^. The use of such alleles in rice breeding is impractical due to these drawbacks. Conversely, mild hypomorphic alleles of *FZP* provide a desirable balance between spikelet and branch development, enhancing both without causing degeneration^24,29,30^. Fujishiro et al.^30^ used a backcrossing strategy to introduce the *qSrn7*/*FZP* (a mild hypomorphic variant of *FZP*) from Kasalath into the Koshihikari cultivar, resulting in a 40–60% increase in yield. Wang et al.^24^ improved the yield of rice variety, TN13 by 10.9% through marker-assisted backcrossing strategy to introduce the *qSBN7* (a mild hypomorphic variant of *FZP*) from IR65598-112-2. These findings underscore the value of utilizing these mild hypomorphic alleles of *FZP* in developing new elite cultivars.

Our previous study highlighted that the *qSBN7* allele displays distinct panicle architectures in different genetic backgrounds^24^. In the *japonica* genetic backgrounds of NIL_TN13^*sbn*^, BSI325, IR76904-7-19, and IR65598-112-2, *qSBN7* exhibits a phenotype resembling that of mild hypomorphic mutants. However, in the *indica* genetic background of BC5_TCS10^*sbn*^, it showed a phenotype analogous to severe hypomorphic mutants^24^. This difference in phenotype is likely attributed to specific gene–gene interactions of *qSBN7* in these different genetic backgrounds. Thus, understanding the underlying mechanisms of spikelet degeneration in BC5_TCS10^*sbn*^ is pivotal for the effective application of the *qSBN7* allele in a broad spectrum of rice breeding programs.

The present study successfully identified three genomic regions from IR65598-112-2, namely IG2, IG4, and IG7, that can effectively reduce the occurrence of degenerated spikelets in BC3_TCS10^*sbn*^. Individually, IG2, IG4, and IG7 reduced the ratio of degenerated spikelets by 32.3%, 17.1%, and 15.7%, respectively (Fig. 5). When combined, these segments resulted in a remarkable 68.1% reduction in degenerated spikelets (Fig. 5). Our comprehensive analysis indicates that each segment harbored QTLs that regulated *FZP* expression, consequently diminishing the number of tertiary branches and the ratio of degenerated spikelets. Previous studies have established a strong correlation between *FZP* expression levels, the number of secondary branches, and the occurrence of degenerated spikelets^24^. In the genetic background of TN13, an increase in secondary branches and spikelets without significant spikelet degeneration, was observed when *FZP* expression reached over 40% of the wild-type level. However, pronounced spikelet degeneration occurred when *FZP* expression fell below 30% of the wild-type level^24^. In this study, BC5_TCS10^*sbn*^ exhibited *FZP* expression levels of approximately 1.8% of the wild-type (TCS10) (Fig. 4f), which was associated with severe spikelet degeneration (Fig. 1). Conversely, BC3_TCS10^*sbn*^, which had less spikelet degeneration, exhibited *FZP* expression levels at approximately 39% of TCS10 (Fig. 4). Additionally, the study observed a significant increased in *FZP* expression and a decreased in the number of tertiary branches in IG2, IG4, and IG7 compared with BC5_TCS10^*sbn*^ (Fig. 5). Therefore, these observations suggest that these genomic regions harbored QTLs that influenced *FZP* expression. Elevated *FZP* expression in BC3_TCS10^*sbn*^ appears to reduce the number of tertiary branches, ultimately decreasing the number of degenerated spikelets. In previous studies, two genes, *OsBZR1* and *OsARF6*, have been identified as regulators of *FZP* expression^29,33^. Comparing their physical map positions on the rice chromosome, only *OsBZR1* is within the IG2 segment. Nonetheless, no synonymous substitutions were detected in the coding region of *OsBZR1* between TCS10 and IR65598-112-2, suggesting other mechanisms may be at play in modulating *FZP* expression through these chromosomal segments.

This study observed a strong correlation between *FZP* expression levels and reduced spikelet degeneration. However, factors related to the efficiency of carbohydrate biosynthesis, distribution, and transfer might also contribute to the decrease in spikelet degeneration in BC3_TCS10^*sbn*^. In BC3_TCS10^*sbn*^, CSSL2, and CSSL7, we observed a significantly wider panicle culm compared with BC5_TCS10^*sbn*^ (Fig. 5j). Studies have indicated that wider culms are often associated with higher carbohydrate transfer efficiency, and plants with wider culms typically have a higher number of grains per panicle^36–40^. Although we observed a negative correlation between culm diameter and spikelet degeneration, varieties with wider culm diameters exhibited lower levels of spikelet degeneration. Whether there is a causal relationship between culm diameter and spikelet degeneration remains to be uncovered.

The number of grains per panicle is the most crucial factor in determining rice yield. To increase grain yield, breeders have been selecting rice varieties with more secondary branches and grain numbers. In this study, BC3_TCS10^*sbn*^ was found to have a significant increase in normal spikelets per panicle compared to TCS10. However, there was no significant increase in panicle weight in BC3_TCS10^*sbn*^ compared to TCS10 (Supplementary Figure S2). The expression level of *FZP* in rice can affect spikelet number per panicle, but an increase in spikelet number per panicle does not necessarily lead to an increase in final rice yield. When spikelet number per panicle is increased in rice, it often leads to trade-offs among yield components^7,8,24,29^. Our previous study showed that the NIL_TN13^*sbn*^ carrying *qSBN7* increased spikelet number per panicle but reduced 1000-grain weight and fertility rate^24^. In the present study, the BC3_TCS10^*sbn*^ carrying *qSBN7* only reduced 1000-grain weight compared to TCS10, while there was no significant difference in fertility rate (Supplementary Figure S2). It is worth noting that varieties with high grain numbers often exhibit lower fertility rates, which may be related to ethylene biosynthesis^41,42^. The *qSBN7* exhibits different trade-off characteristics in different varieties, whether this phenomenon is related to ethylene regulation under different genetic backgrounds needs to be further confirmed.

Our study reveals that the combination of three minor chromosomal substitution segments significantly influenced panicle architecture differences between the two BILs. However, even with the pyramiding of these three segments in BC3_TCS10^*sbn*^, we still observed a 10–40% rate of spikelet degeneration or replacement by continuous bract-like structures (Fig. 5i and Table 1). This suggests the presence of additional QTL differences between IR65598-112-2 and BC3_TCS10^*sbn*^ that may affect spikelet degeneration. In future studies, we aim to conduct fine mapping and RNA-seq analysis to further uncover why *qSBN7* causes spikelet degeneration in BC5_TCS10^*sbn*^. This will contribute to the widespread application of *qSBN7* in rice breeding programs to enhance grain yield.

## Materials and methods

### Plant materials

Through backcross breeding, we developed a BC_5_-derived BIL (BC5_TCS10^*sbn*^), which have the homozygous *qSBN7* allele of IR65598-112-2 cultivars, in the genetic background of *indica* cultivar TCS10^24^ (previously named NIL_TCS10^*sbn*^). To obtain a BIL with reduced spikelet degeneration, a BC_3_F_2_ population derived from the same recurrent and donor parent was developed. Three BC_3_F_2_ individuals with minor panicle degeneration were selected and self-pollinated twice to produce three BC_3_F_4_ BILs. After visual evaluation, we chose one BIL, BC3_TCS10^*sbn*^, which carries the homozygous *qSBN7* alleles of IR65598-112-2 and exhibits slight panicle degeneration, for further experiments.

To develop three CSSLs, we crossed the BC3_TCS10^*sbn*^ with BC5_TCS10^*sbn*^. Three CSSLs, CSSL2 (with a homozygous introgression segment on chromosome 2), CSSL4 (with a homozygous introgression segment on chromosome 4), and CSSL7 (with a homozygous introgression segment on chromosome 7), were developed by screening 260 BC_4_F_2_ plants using seven Kompetitive Allele Specific PCR (KASP) markers (Supplementary Table S1) located at the introgression segments of IR65598-112-2 in BC3_TCS10^*sbn*^. The KASP genotyping master mix was supplied by LGC Genomics (Middlesex, UK). The KASP analysis was carried out according to the manufacturer’s protocol. The breeding process for our plant materials is detailed in Supplementary Fig. S3.

### Growing conditions and phenotypic evaluation

In the current study, fifty-two plants from each line were grown in paddy fields in Chia-Yi, Taiwan (23°42’N, 120°28’E), with a spacing of 20 cm between plants and 30 cm between rows. The rice plants were cultivated in a well-irrigated paddy field according to conventional management practices. Each field received 160 kg ha^−1^ of nitrogen fertilizer.

To assess the yield components of TCS10 and BC3_TCS10^*sbn*^, each line was cultivated with three replications. At maturity, several traits were evaluated, such as the number of secondary branches per panicle, the number of normal spikelets per panicle, the percentage of filled grains, 1000-grain weight, and panicle weight. Six individuals from each line were randomly selected for measurement. Differences between the lines were analyzed using Student's *t* test.

For phenotypic assessment of panicle-related and source-related traits, we selected sixteen individuals from the middle of a row for each line. We measured panicle-related traits on the main panicles, identified as the tallest tillers of each plant. Panicle-related traits were measured by manually counting. In the present study, we categorized all observed degenerated organs, including degenerated spikelets and bract-like structures on branches, as degenerated spikelets. The number of degenerated spikelets and the ratio of degenerated spikelets of the panicle were analyzed using the following formulae.

The number of degenerated spikelets = Number of degenerated spikelets + Number of continuous bract-like structures.

The ratio of degenerated spikelets = 100×(Number of degenerated spikelets + Number of continuous bract-like structures)/(Number of normal spikelets + Number of degenerated spikelets + Number of continuous bract-like structures).

The diameter of the rachis base of these main panicles served as the measure for culm diameter. We evaluated chlorophyll content using a Soil Plant Analysis Development chlorophyll meter (Konica–Minolta, Osaka, Japan), adhering to the instructions in its operation manual.

### Whole genome sequencing and analysis

To investigate the genetic backgrounds of BC3_TCS10^*sbn*^ and BC5_TCS10^*sbn*^, the whole genome DNA libraries of TCS10, IR65598-112-2, BC3_TCS10^*sbn*^, and BC5_TCS10^*sbn*^ were constructed and sequenced as 150-bp pair-end reads at Genomics (Taipei, Taiwan). Around 10 Gb of raw reads were obtained each on an Illumina NovaSeq 6000 platform. Paired-end reads were trimmed to remove adapters and low-quality bases (< 20) and filtered for reads < 100-bp using Trimmomatic 0.36^43^. Filtered short reads were then mapped to the Nipponbare reference genome sequences IRGSP1.0^44^ using BWA-MEM^45^. Single nucleotide polymorphisms (SNPs) were called for the entire data set of TCS10, IR65598-112–2, BC3_TCS10^*sbn*^, and BC5_TCS10^*sbn*^ using the mpileup command of SAMtools^46^. SNPs were filtered using the filter command of bcftools^46^ according to the following criteria: quality score (QUAL) > 30.0 and depth (DP) > 20 across all samples.

### RNA isolation and quantitative PCR analysis

For RNA extraction, we harvested inflorescences at the 1 mm stage of development. Three inflorescences from a single plant were collected as one biological replicate. We extracted total RNA using the RNeasy Plant Mini Kit (QIAGEN, Valencia, CA) with RNase-free DNase I (QIAGEN, Valencia, CA). To evaluate the expression level of *FZP*, we synthesized complementary DNA (cDNA) from 0.6 μg of total RNA using the iScrip cDNA Synthesis Kit (Bio-Rad, Hercules, CA). The transcriptional levels of *FZP* were quantified through qPCR analysis. Each assay incorporated three biological and three technical replicates. The qPCR was performed on a CFX96 Connect Real-Time PCR detection system (Bio-Rad). We selected the rice ubiquitin gene *UBQ5* (*LOC_Os01g22490*) as the internal control. The primers and probe are described in a previous study^24^.

### Supplementary Information

Supplementary Figures.

Supplementary Table 1.

## Data Availability

The raw DNA sequence data is available in the NCBI Sequence Read Archive with accession number PRJNA1086197.
