# Supplementary material for: Identification of genomic regions controlling spikelet degeneration under FRIZZLE PANICLE (FZP) defect genetic background in rice
Source: Sci Rep. 2024 May 30;14:12451. doi: 10.1038/s41598-024-63362-8 (PMC11139880; doi:10.1038/s41598-024-63362-8)
Supplement: Supplementary file 1 — Supplementary Figures. [file 41598_2024_63362_MOESM1_ESM.doc]

**
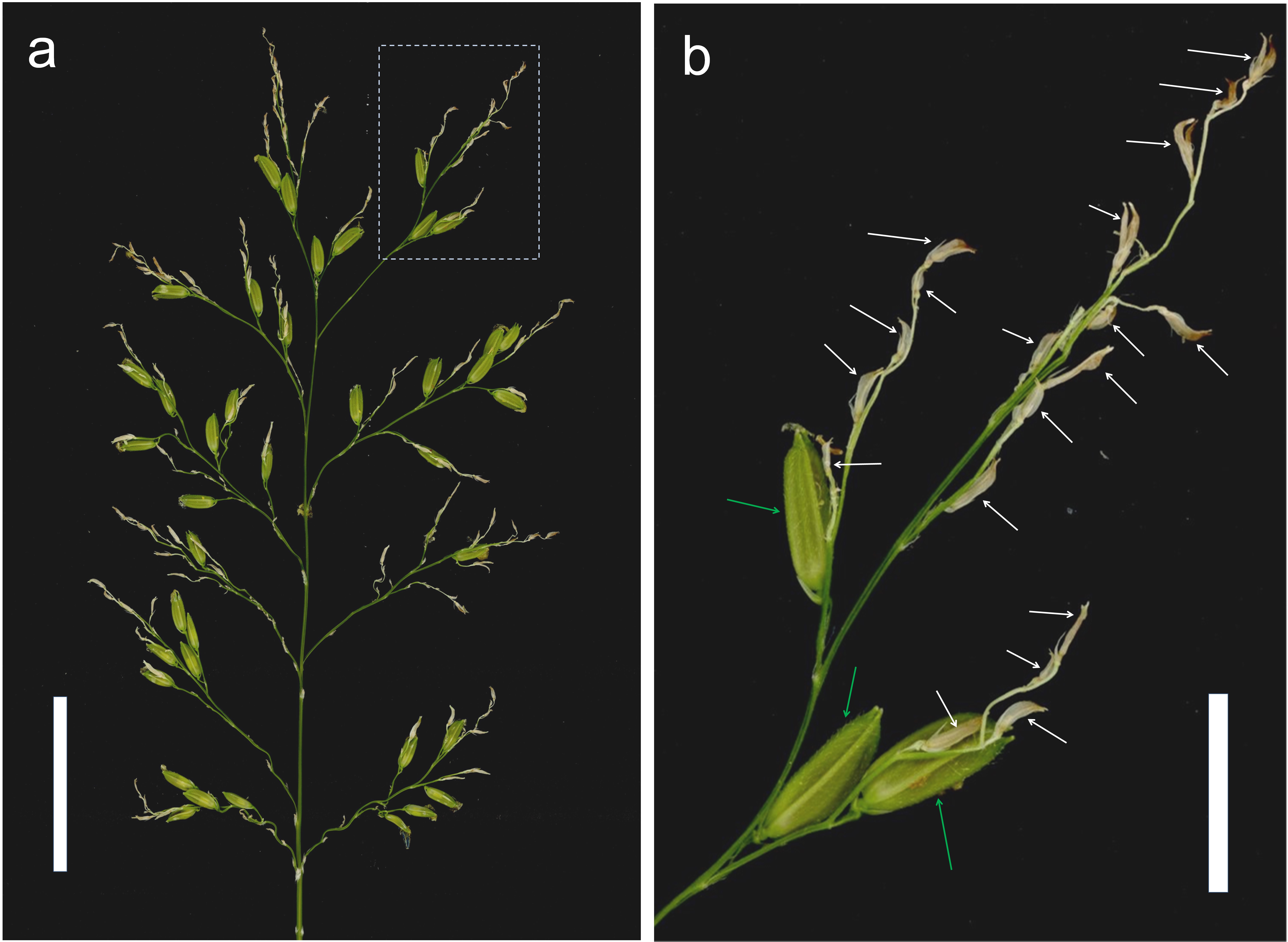
**

**Supplementary Figure S1.** Panicle structure of BC5_TCS10*^sbn^* at the heading stage. (**a**) Panicle structure of BC5_TCS10*^sbn^*. Scale bar, 4 cm. (**b**) Normal spikelets (green arrows), degenerated spikelets (white arrows) were observed in the panicle of BC5_TCS10*^sbn^*. Scale bar = 10 mm.


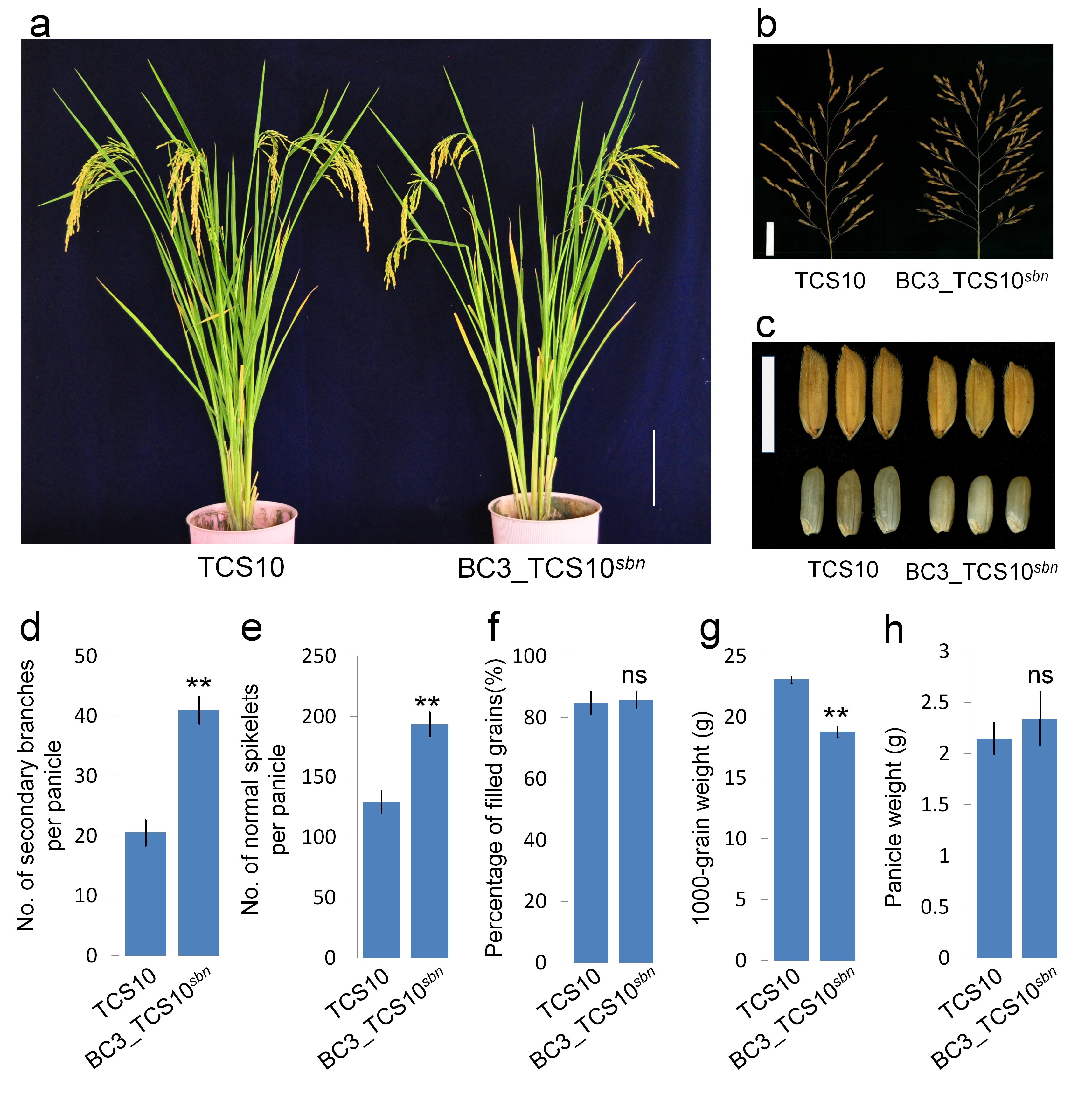


**Supplementary Figure S2.** Phenotypic characterization of TCS10 and BC3_TCS10*^sbn^*. (**a**) Plant structure of TCS10 and BC3_TCS10*^sbn^*. Bar, 20 cm. (**b**) Panicle structure of TCS10 and BC3_TCS10*^sbn^*. Bar, 4 cm. (**c**) Grain size of TCS10 and BC3_TCS10*^sbn^*. Bar, 10 mm. (**d**) Number of secondary branches per panicle. (**e**) Number of normal spikelets per panicle. (**f**) Percentage of filled grains (%). (**g**) 1000-grain weight (gram). (**h**) Panicle weight (gram). A completely randomized design with three replications was used in the trial. Values in (**d–h**) are means ± SD (n = 6 plants). Student’s *t*-test was used to examine *P*-values. **Significant at 1% level; ns, not significant; No, number. Percentage of filled grains = 100 × (number of filled grains per panicle) / (number of normal spikelets per panicle).


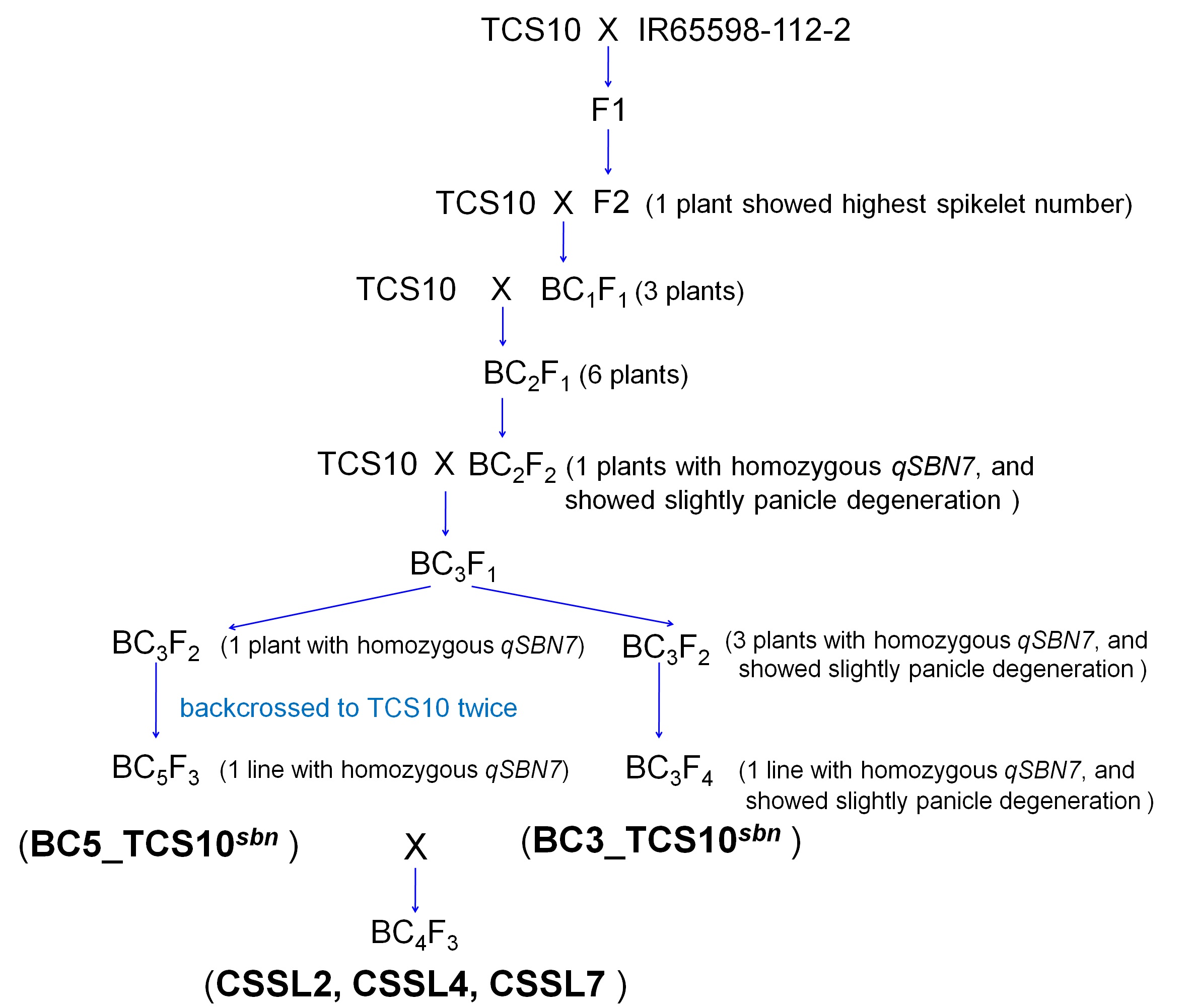


**Supplementary Figure S3.** Development of genetic material in this study.
