# Supplementary material for: Identification of genomic regions controlling spikelet degeneration under FRIZZLE PANICLE (FZP) defect genetic background in rice
Source: Sci Rep. 2024 May 30;14:12451. doi: 10.1038/s41598-024-63362-8 (PMC11139880; doi:10.1038/s41598-024-63362-8)
Supplement: Supplementary file 2 — Supplementary Table 1. [file 41598_2024_63362_MOESM2_ESM.doc]

**Supplementary Table S1** Competitive allele-specific PCR (KASP) markers used in this study

| Assays ID | Primer_AlleleFAM | Primer_AlleleHEX | Primer_Common |
| --- | --- | --- | --- |
| TCS10_C2_11 | TCATGGCGATAGATCAAAGCCT | CATGGCGATAGATCAAAGCCC | AAGGGGTGGTTTAGTAATTGGA |
| TCS10_C2_29 | CAACCGCCACCATTCTACCA | CAACCGCCACCATTCTACCC | AGAACACAACGATGGCAAATAAC |
| TCS10_C4_1 | GCGTGTAGGCAAATCTACTCAACA | GCGTGTAGGCAAATCTACTCAACT | CTAAACATTCTCGAAAGGTCCG |
| TCS10_C4_20 | CCACATCTATCTTGCCTCCACT | CCACATCTATCTTGCCTCCACC | AGGGAATGTTGTGAAGCTTACC |
| TCS10_C7_9 | AGTAGCCCTCTAGCTCTGACTATGC | ACAGTAGCCCTCTAGCTCTGACTATGT | TTGCTTACATTTTTGGCCTACAC |
| TCS10_C7_26 | CCTGGCCTGTTGTCTCACC | GCCTGGCCTGTTGTCTCACT | ATATGCGGTCGGTTACTTCCT |
| TCS10_C7_28 | GTCCTGTCAAAATTTTCCGGTCTTTT | GTCCTGTCAAAATTTTCCGGTCTTTC | CAGGTAATGTCAAGGATACGATCATATCTT |
